# Identification of immune-associated genes in diagnosing osteoarthritis with metabolic syndrome by integrated bioinformatics analysis and machine learning

**DOI:** 10.3389/fimmu.2023.1134412

**Published:** 2023-04-17

**Authors:** Junchen Li, Genghong Wang, Xilin Xv, Zhigang Li, Yiwei Shen, Cheng Zhang, Xiaofeng Zhang

**Affiliations:** ^1^ The Graduate School, Heilongjiang University of Chinese Medicine, Harbin, China; ^2^ The Third Affiliated Hospital of Heilongjiang University of Chinese Medicine, Harbin, China; ^3^ Teaching and Research Section of Orthopedics and Traumatology, Heilongjiang University of Chinese Medicine, Harbin, China; ^4^ The Second Department of Orthopedics and Traumatology, The Second Affiliated Hospital of Heilongjiang University of Chinese Medicine, Harbin, China; ^5^ The Bone Injury Teaching Laboratory, Heilongjiang University of Chinese Medicine, Harbin, China

**Keywords:** differentially expressed genes, osteoarthritis, metabolic syndrome, machine learning, immune infiltration

## Abstract

**Background:**

In the pathogenesis of osteoarthritis (OA) and metabolic syndrome (MetS), the immune system plays a particularly important role. The purpose of this study was to find key diagnostic candidate genes in OA patients who also had metabolic syndrome.

**Methods:**

We searched the Gene Expression Omnibus (GEO) database for three OA and one MetS dataset. Limma, weighted gene co-expression network analysis (WGCNA), and machine learning algorithms were used to identify and analyze the immune genes associated with OA and MetS. They were evaluated using nomograms and receiver operating characteristic (ROC) curves, and finally, immune cells dysregulated in OA were investigated using immune infiltration analysis.

**Results:**

After Limma analysis, the integrated OA dataset yielded 2263 DEGs, and the MetS dataset yielded the most relevant module containing 691 genes after WGCNA, with a total of 82 intersections between the two. The immune-related genes were mostly enriched in the enrichment analysis, and the immune infiltration analysis revealed an imbalance in multiple immune cells. Further machine learning screening yielded eight core genes that were evaluated by nomogram and diagnostic value and found to have a high diagnostic value (area under the curve from 0.82 to 0.96).

**Conclusion:**

Eight immune-related core genes were identified (*FZD7*, *IRAK3*, *KDELR3*, *PHC2*, *RHOB*, *RNF170*, *SOX13*, and *ZKSCAN4*), and a nomogram for the diagnosis of OA and MetS was established. This research could lead to the identification of potential peripheral blood diagnostic candidate genes for MetS patients who also suffer from OA.

## 1 Introduction

Osteoarthritis (OA) is one of the common degenerative diseases of the musculoskeletal system in orthopedics, often involving one or more joints, and has a high prevalence worldwide (1), and according to WHO, there is a 10% chance of OA occurring in people over 60 years of age worldwide (2). So far, the causative factors and pathogenesis of OA are not clear, but most scholars believe that its pathogenesis is a combination of several factors, such as obesity, aging, trauma, excessive joint strain, metabolic disorders, inflammation, and genetics (3).

Metabolic syndrome (MetS) is a metabolic disorder characterized by abdominal obesity, dyslipidemia, hyperglycemia, and hypertension (4). According to the National Institute for Health and Nutrition Examination Survey, around one in five adults in the United States suffer from MetS (5). Most patients with metabolic syndrome are associated with varying degrees of obesity, which increases stress on the entire musculoskeletal system and poses a higher risk for the development of osteoarthritis and various other musculoskeletal disorders. Osteoarthritis and metabolic syndrome share common mechanisms of inflammation, oxidative stress, and metabolic dysfunction in their etiology (6). Traditionally, osteoarthritis is a non-inflammatory disease affected by trauma or metabolic dysregulation, and age-related joint degeneration is thought to be a causal factor in the development of the disease (7, 8). However, a growing body of evidence suggests that low-grade inflammation may be a key factor driving the pathogenesis of OA (9). Inflammation and metabolic disorders play a very important role in the progression of osteoarthritis (10, 11). Risk factors, including diabetes, hypertension, and hyperlipidemia, are largely involved in osteoarthritis through the release of inflammatory and adipokines that accelerate the progression of osteoarthritis by driving articular cartilage degeneration and bone marrow lesions (12). Several theories describe how MetS risk factors affect the progression of OA, such as high blood pressure can cause subchondral ischemia, abnormal lipids can cause lipid deposition in chondrocytes, and high blood glucose can cause oxidative stress and low inflammation, eventually leading to cartilage destruction (13). In addition, central obese patients also have abnormal leptin and lipocalin levels, which can further aggravate the development of OA (6, 14). To date, metabolic syndrome-associated osteoarthritis (MetS-OA) has been well characterized as a distinct phenotype of osteoarthritis (15, 16), and the goal of this model is to investigate the relationship between inflammatory response and metabolic disorders in order to improve the concept of treating MetS and to aid in the reduction of inflammatory response in OA patients. There is some evidence to support this, but the mechanism of action in metabolic syndrome-related osteoarthritis is still being investigated. In recent years, comprehensive bioinformatics analysis has been used to identify novel genes associated with various diseases that can serve as diagnostic and prognostic biomarkers. However, the common diagnostic and interlinked genes of OA and MetS are not known. Therefore, this study used a bioinformatics approach to screen for biomarkers associated with immune infiltration in both, which could help identify immune-related potential diagnostic markers for OA in patients with MetS.

## 2 Materials and methods

### 2.1 Microarray data

**Figure 1:**
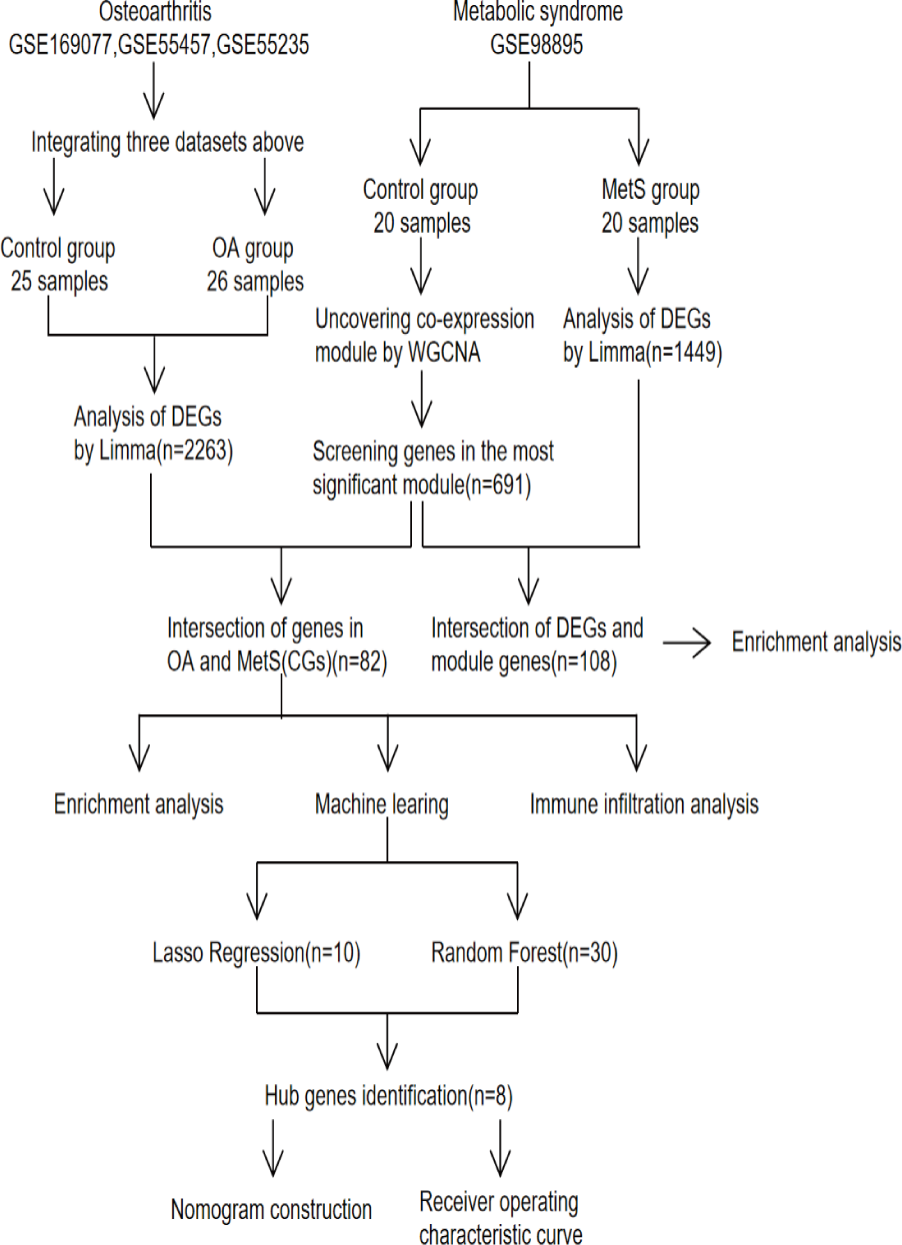
Study flowchart, GSE, gene expression omnibus series; WGCNA, weighted gene co-expression network analysis; Limma, linear models for microarray data; DEGs, differentially expressed genes.

The datasets used were all from the NCBI Gene Expression Omnibus (GEO) database (17), and the OA datasets were GSE169077, GSE55457, and GSE55235 (18); while the MetS dataset was GSE98895 (19). **Figure 1** depicts the study flowchart.

### 2.2 Data processing and differential gene screening

The following steps were all carried out using the R software (https://www.bioconductor.org/), starting with “affy” to calibrate, log-transform, and normalize the three OA datasets, then merging them and using “SVA” to remove batch effects, with p-values set to 0.05 and |log2 fold change (FC)|>1.5, and “limma” for differential gene screening. **Table 1** presents detailed dataset information, including the microarray platform, sample groups, and numbers.

**Table 1:** Basic information of GEO datasets used in the study.

GSE series	Type	Sample size		Platform
		Control	Osteoarthritis	
GSE169077	RNA	5	6	GPL96
GSE55457	RNA	10	10	GPL96
GSE55235	RNA	10	10	GPL96
		**Control**	**Metabolic** **syndrome**	
GSE98895	RNA	20	20	GPL6947

### 2.3 Weighted gene co-expression network analysis

WGCNA (20) was used to investigate the gene modules most closely linked to MetS. First, the top 50% of genes with the highest median absolute deviation (MAD) were filtered. The expression matrix was then filtered to remove ineligible data. Third, use a “soft” threshold power (β) for co-expression of similarity to calculate adjacency. Then, using dynamic tree cuts and hierarchical clustering, a topological overlap matrix (TOM) was created to group genes into modules by random colors, and a gene dendrogram was constructed using a TOM-based measure of phase dissimilarity and a minimum gene cluster size (n=100). Fifth, for the next step of the study, the dissimilarity of the module genes was calculated, and the average linkage hierarchy clustering was performed. It is finally visualized.

### 2.4 Functional enrichment analysis

Sangerbox (http://www.sangerbox.com/tool) was used for Gene Ontology (GO) (21) and Kyoto Encyclopedia of Genes and Genomes (KEGG) (22) enrichment analysis.

### 2.5 Machine learning

Machine learning algorithms are used to screen the core genes for OA diagnosis. “glmnet” (23) was used for LASSO (24) regression, and “randomForest” (25) was used for RF (26) analysis. The two intersecting genes were used as the core genes for OA diagnosis.

### 2.6 Nomogram construction and evaluation of recipient operating characteristics

Nomogram was created for the screened genes using the “rms” (27) R package, and its value in the clinic was determined by the area under the curve (AUC) and 95% CI using ROC. When the AUC exceeds 0.7, it is considered to have diagnostic value.

### 2.7 Immune infiltration analysis

The CIBERSORT algorithm is used to determine the proportion of immune cells in cells or tissues (28). The bar graphs show the proportion of each type of immune cell in various samples, and the “corrplot” (29) R package is used to generate a heat map of the correlation between 22 immune cells. The vioplot was used to visualize the differences between the OA and normal immune cell groups.

### 2.8 Statistical analysis

R software version 4.2.2 and Sangerbox were used for all statistical analyses. To compare normally distributed continuous variables between two groups, the Independent Student’s t-test was used, and the Wilcoxon rank sum test was used to analyze non-normally distributed variables. Every statistical test was two-sided. The statistical significance level was set at p-Value <0.05.

## 3 Results

### 3.1 Differentially expressed genes

**Figure 2:**
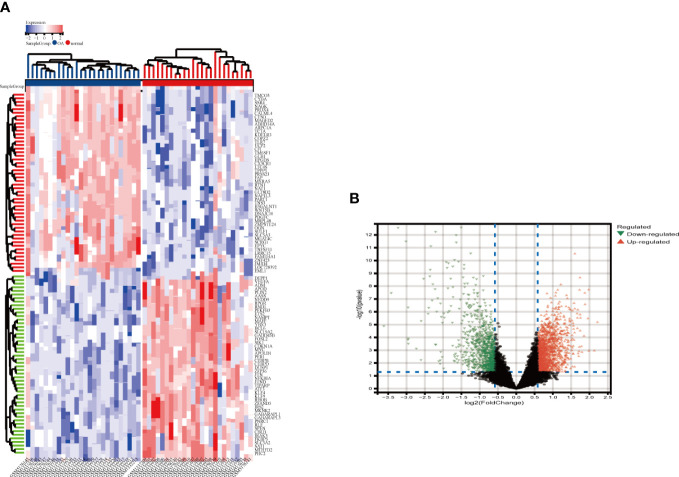
Heatmap and valcano plot for the DEGs identified from the integrated OA dataset. **(A)** Each row shows the DEGs, and each column refers to one of the samples of OA cases or controls. The red and blue represent DEGs with upregulated and downregulated gene expression, respectively. **(B)** Red and green plot triangles represent DEGs with upregulated and downregulated gene expression, respectively. OA, Osteoarthritis: DEGs, differentially expressed genes.

**Figure 3:**
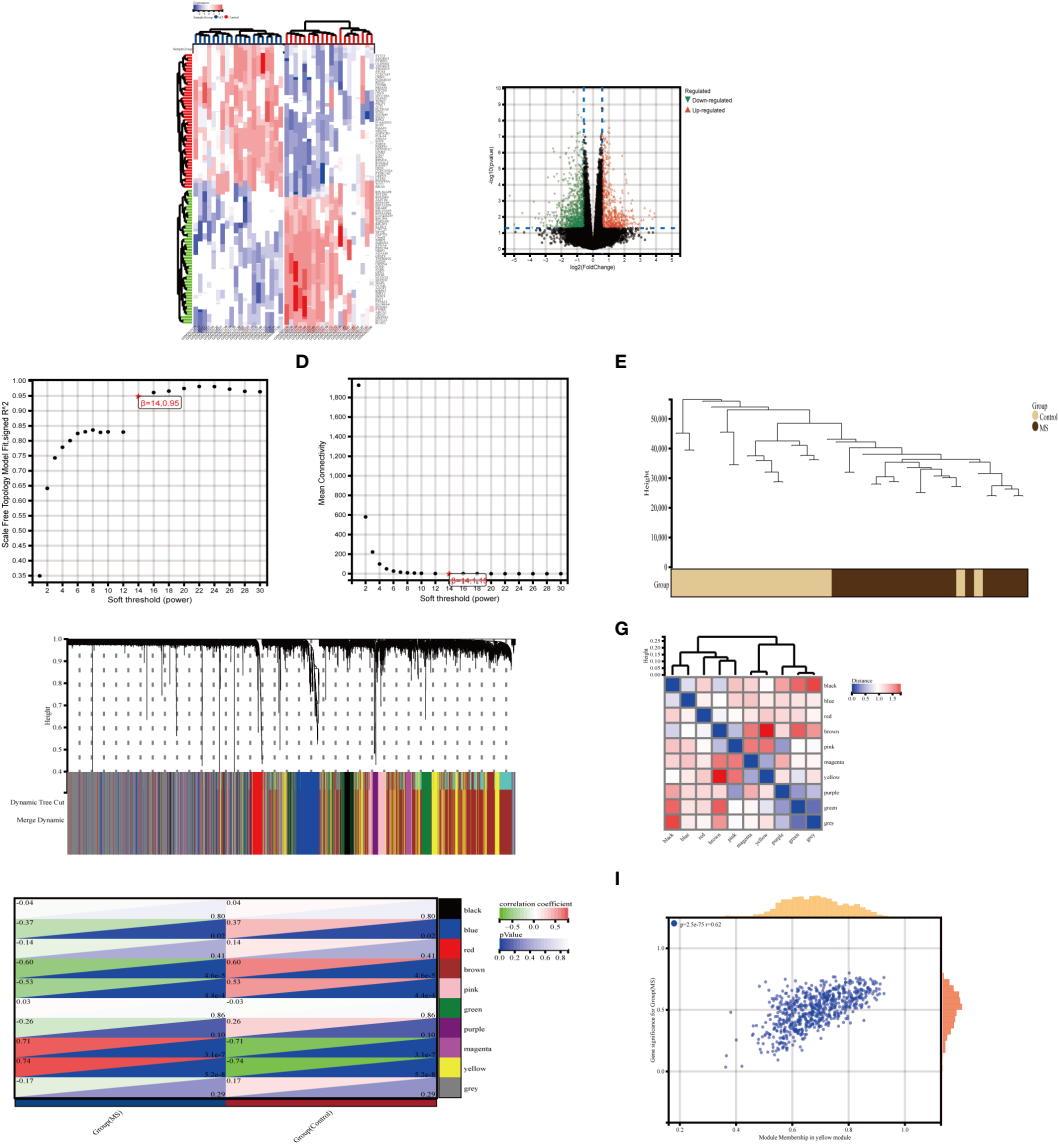
Identification of DEGs via Limma and module genes via WGCNA in MetS. **(A)** The heatmap displays the top 50 upregulated and downregulated DECs identified from MetS dataset. Each row represents the intersection of genes, and each column represents one of MetS cases or controls. Red and blue represent upregulated and downregulated ge expression. **(B)** The volcano plot shows all DEGs, of which red and green triangles refer to significant DEGs. **(C, D)** b= 14 is selected as the soft threshold with the combined analysis of scale independence and average connectivity. **(E)** Clustering dendrogram of the MetS and control samples. **(F)** Gene co-expression modules represented by different colors under the gene tree **(G)** Heatmap of eigengene adjacency. **(H)** Heatmap of the association between modules and Mers. The yellow module is shown to be correlated significantly with MetS. Numbers at the top and bottom brackets represent the correlation coefficient and p-value, respectively. **(I)** Correlation plot between module membership and gene significance of genes included in the yellow module WGCNA, weighted gene co-expression network analysis, Limma, linear models for microarray data; DEGs, differentially expressed genes.

The integrated OA dataset yielded a total of 2263 DEGs after LIMMA analysis, with 1341 up-regulated genes and 922 down-regulated genes. **Figures 2A, B** show the heat map and volcano map generated from the above data. Furthermore, the MetS dataset yielded 1449 DEGs, including 605 up-regulated genes and 844 down-regulated genes (**Figures 3A, B**).

### 3.2 Selection of key modules

In MetS, use WGCNA to filter critical modules. When the soft threshold =14, the scale-free network performs best (**Figures 3C, D**). **Figure 3E** depicts the clustering tree graph for both groups and the ten randomly colored gene modules obtained (**Figures 3F, G**). The yellow module (691 genes) had the highest MetS correlation (**Figure 3H**) and can be used as a key module in the following analysis. **Figure 3I** shows the results of a correlation analysis for the genes in the yellow module, which showed a positive correlation (r=0.62).

### 3.3 Functional enrichment analysis of metabolic syndrome

**Figure 4:**
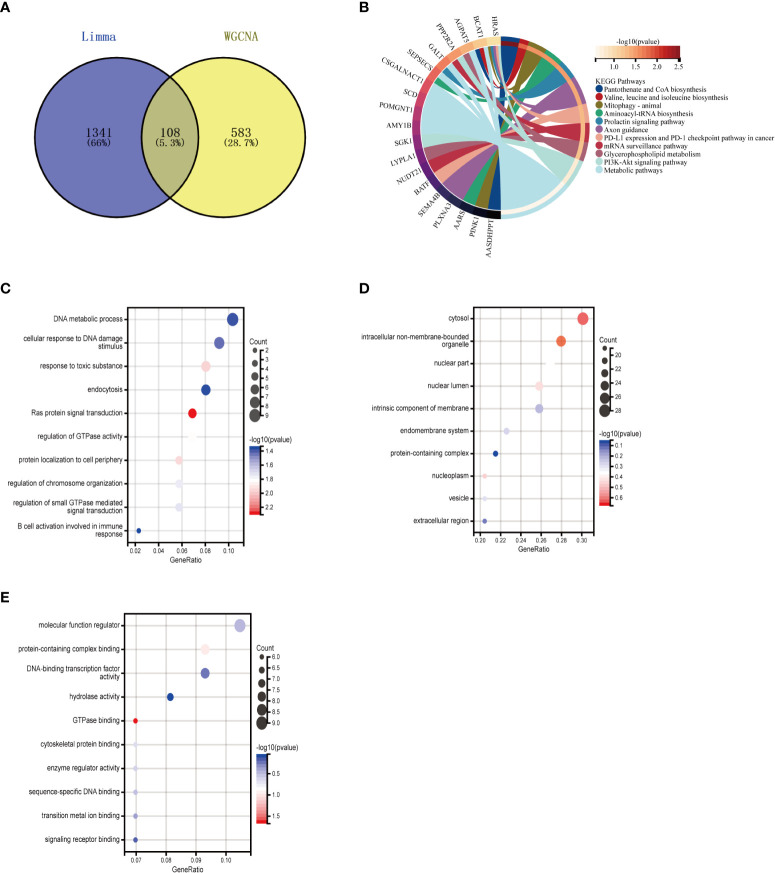
Enrichment analysis of the intersection of genes in MetS. **(A)** Venn diagram shows that 108 genes are identified from the intersection of DEGs via Limma and yellow module genes via WGCNA. **(B)** KEGG pathway analysis of the intersection of genes. Different colors represent various significant pathways and related enriched genes. **(C–E)** GO analysis of the Intersection of genes, including biological process, cellular component, and molecular function, respectively. The y-axis represents different GO terms, the x-axis represents gene ratio enriched in relative GO terms, the circle size refers to gene numbers, and the color represents p-value. MetS, Metabolic syndrome: KEGG, Kyoto Encyclopedia of Genes and Genomes GO, Gene Ontology: WGCNA, weighted gene co-expression network analysis: Limma, linear models for microarray datac DEGs, differentially expressed genes.

The intersection of 1449 DEGs and 691 modular genes yielded a total of 108 genes (**Figure 4A**). The “PI3K-Akt signaling pathway” and “glycerophospholipid metabolism” were primarily enriched in KEGG analysis (**Figure 4B**). According to GO analysis, the main enrichment in biological process (BP) terms was in “B cell activation involved in immune response” and “cellular response to DNA damage stimuli” (**Figure 4C**). It is primarily found in “cell membrane,” “nuclear lumen,” and “vesicles” in terms of cellular component (CC) ontology (**Figure 4D**). It was primarily enriched in “molecular function regulators,” “enzyme regulator activity,” and “signaling receptor binding” in molecular function (MF) analysis (**Figure 4E**).

### 3.4 Enrichment analysis of osteoarthritis with metabolic syndrome

**Figure 5:**
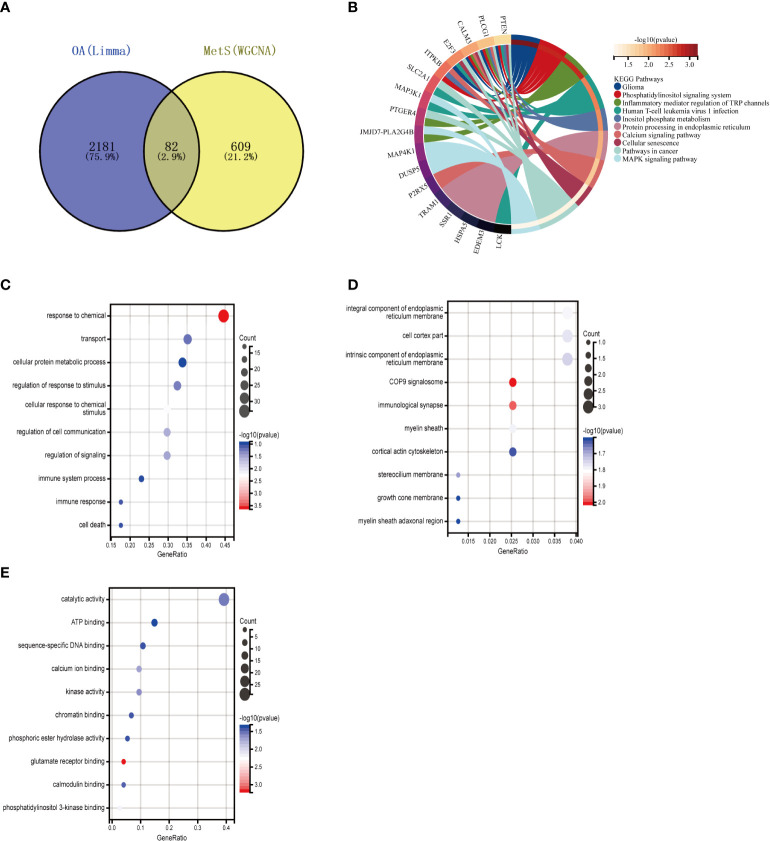
Enrichment analysis of common genes from OA with MetS. **(A)** Venn diagram shows that 82 common genes are identified from the intersection of genes in OA using Limma and MetS using WGCNA. **(B)** KEGG analysis of 82 common genes. **(C–E)** GO analysis (hiological process, cellular component, and molecular function) of 82 common genes. OA. Osteoarthritis: MetS, Metabolic syndrome, WGCNA, weighted gene co-expression network analysis.

To further explore whether key MetS-related genes might be associated with the pathogenesis of OA, 82 genes were identified by the Venn diagram from the intersection of OA DEGs and MetS key module genes (**Figure 5A**). According to KEGG analysis, 82 genes were primarily enriched in “inflammatory mediator regulation of TRP channels,” “cellular senescence,” and “MAPK signaling pathway,” all of which are closely related to the immune system (**Figure 5B**). GO analysis revealed that they were primarily enriched in “immune response,” “cell death,” and “immune system process” (BP); “integral component of endoplasmic reticulum membrane,” “stereocilium mem-brane” and “immunological synapse,” (CC); and “catalytic activity,” “ATP binding,” and “sequence-specific DNA binding” (MF) (**Figures 5C–E**).

### 3.5 Core genes screening using machine learning

**Figure 6:**
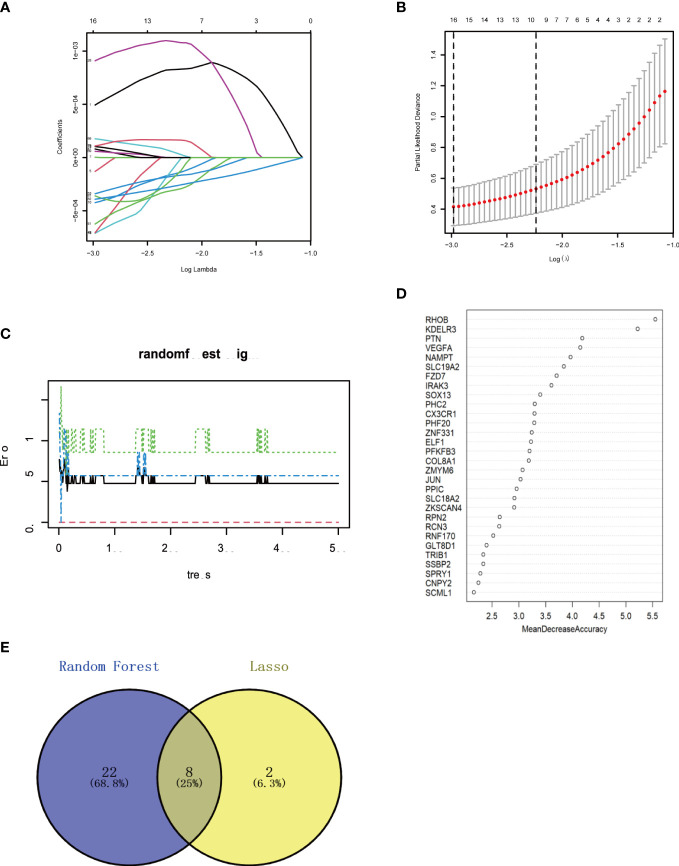
Machine learning in screening candidate diagnostic biomarkers for OA with MetS **(A, B)** Biomarkers screening in the Lasso model. The number of genes (n-10) corresponding to the lowest point of the curve is the most suitable for OA with MetS diamos. **(C, D)** The random forest algorithm shows the error in OA; control group and genes are ranked based on the importance score. **(E)** Venn diagram shows that eight candidate diagnostic genes are identified via the above two algorithms OA. Osteoarthritis: MetS Metabolic syndrome.

The Lasso regression and RF algorithms were used to identify core genes and create relevant nomograms for ROC analysis. Lasso regression screened 10 candidate genes (**Figures 6A, B**), and the RF algorithm identified 30 most important genes (**Figures 6C, D**), and the two were taken to intersect (**Figure 6E**), resulting in the identification of eight genes (FZD7, IRAK3, KDELR3, PHC2, RHOB, RNF170, SOX13, and ZKSCAN4).

### 3.6 Determining diagnostic value

**Figure 7:**
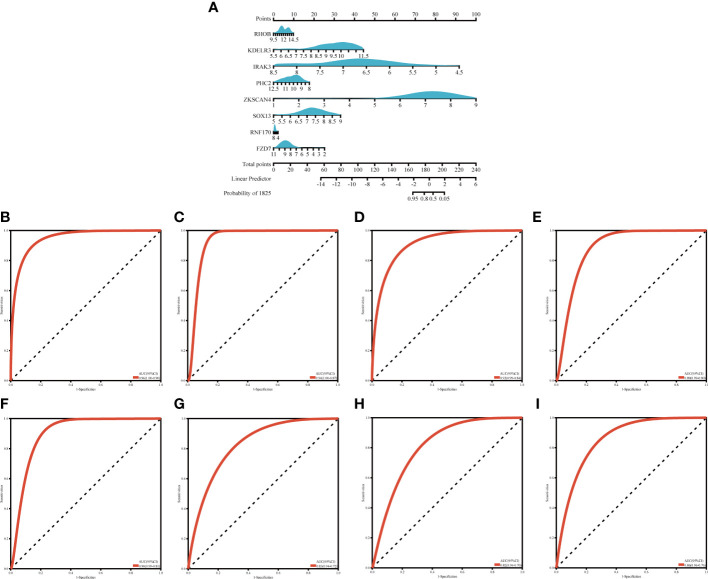
Nogrim comtraction and the diagnostic value evaluation **(A)** The visible nomogram for diagnosing OA with MetS **(B–I)** The ROC curve of each candidate gene (RHOR, KDELR3, TRAKA, PHC2, ZKSCANA, SOX13, RNF 170 and FZD7) and nomogram show the significant OA with MetS diagnostic value. OA. Osteoarthritis, MetS, Metabolic syndrome; AUC, area under the curve.

We created a nomogram (**Figure 7A**) and plotted ROC curves based on the eight candidate genes to assess the diagnostic value of each gene. The calculated AUCs and 95% confidence intervals were as follows: FZD7 (AUC 0.86, CI 0.96–0.76), IRAK3 (AUC 0.92, CI 0.99–0.84), KDELR3 (AUC 0.94, CI 1.00–0.87), PHC2 (AUC 0.89, CI 0.99–0.80), RHOB (AUC 0.96, CI 1.00–0.90), RNF170 (AUC 0.82, CI 0.94–0.70), SOX13 (AUC 0.83, CI 0.94–0.72), ZKSCAN4 (AUC 0.90, CI 0.99–0.81) (**Figures 7B–I**). The findings indicated that the acquired genes had a high value for the diagnosis of OA in combination with MetS.

### 3.7 Immune infiltration analysis

We discovered that genes associated with MetS can also play a role in OA, primarily in immune regulation. An in-depth examination of the nomogram and ROC revealed that they could be used as potential biomarkers for the diagnosis of OA, which was confirmed by immune infiltration.

**Figure 8:**
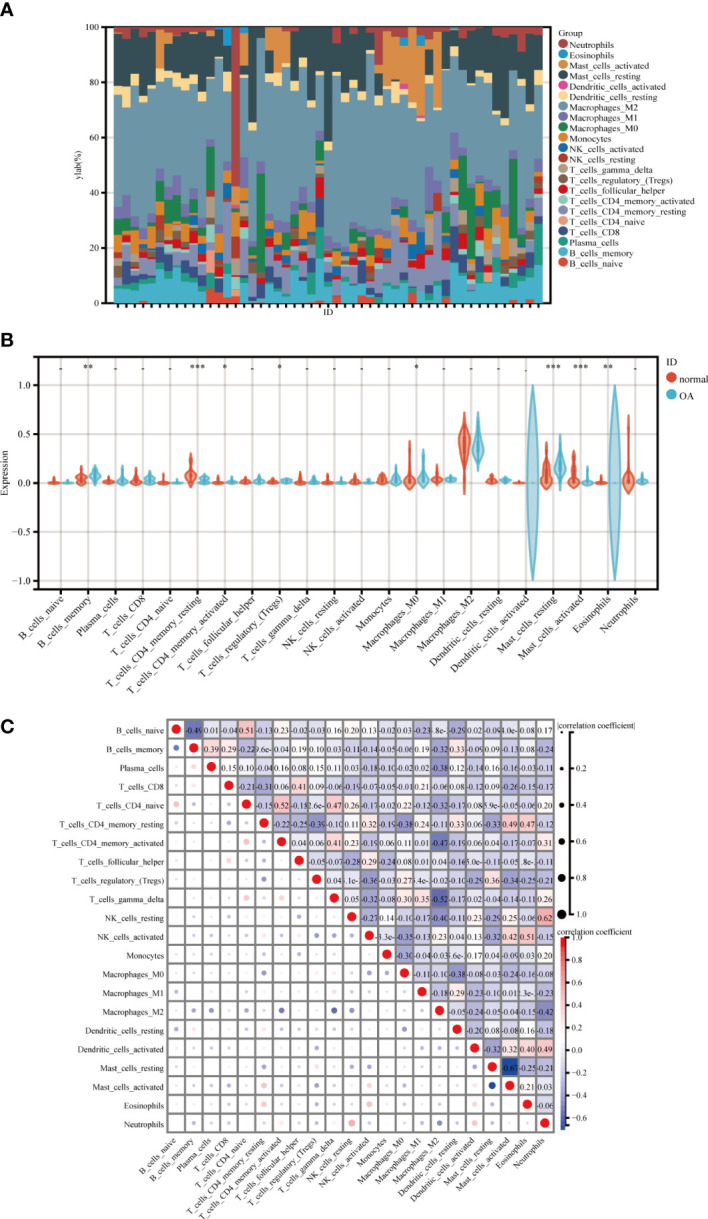
Immune cell infiltration analysis between OA and control. **(A)** The proportion of 22 kinds of immune cells in different samples visualized from the burplot. **(B)** Comparison regarding the proportion of 22 kinds of immune cells between OA and control groups visualized by the vioplot. **(C)** Correlation of 22 immune cell type compositions, *,p < 0.05, **,p < 0.01, ***,p < 0.001. Both horizontal and vertical axes demonstrate immune cell subtypes OA, Osteoarthritis.

The percentage of 22 immune cells in each sample is shown in the bar graph for both datasets (**Figure 8A**). Voltammograms showed higher levels of B cells memory, Macrophages M0, Dendritic cells activated, Mast cells resting and Eosinophils in OA patients, and lower levels of T cells CD4 memory resting, Macrophages M2, Mast cells activated and Neutrophils (**Figure 8B**). Correlation analysis of the 22 immune cell types showed that NK cells resting was positively correlated with Neutrophils (r=0.62), T cells CD4 naive was positively correlated with T cells CD4 memory activated (r=0.52), Mast cells activated was negatively correlated with Mast cells resting (r=-0.67), and Macrophages M2 was negatively correlated with T cells gamma delta (r=-0.52) (**Figure 8C**). In conclusion, OA patients have varying degrees of multiple immune cell infiltration that may be potential regulatory points for the treatment of OA.

## 4 Discussion

OA is one of the most significant causes of disability in middle-aged and older adults and places a significant burden on public health (30). Several biomarkers for OA have been identified in recent studies, including osteoclast protein (OPN), cartilage oligomeric matrix protein (COMP), cartilage acidic protein 1, and CRTAC1 (31, 32). Studies that combine these two diseases are relatively rare. There were currently no markers for diagnosing OA using nomogram and machine learning methods, so we combined both bioinformatics analysis and machine learning and evaluated their diagnostic value using nomogram and ROC. Notably, we identified eight key immune-related candidate genes (*FZD7*, *IRAK3*, *KDELR3*, *PHC2*, *RHOB*, *RNF170*, *SOX13*, and *ZKSCAN4*) and developed a nomogram for the diagnosis of OA in MetS patients.

We were able to approximate the odds of developing OA in MetS patients by simply testing the peripheral blood of MetS patients with known expression of core genes, given that the MetS samples used in this study were all from peripheral blood. Because of its simplicity and effectiveness, peripheral blood testing is used in the diagnosis of many diseases (33, 34). Following that, we will develop a more refined model that can accurately reflect gene expression and assign it a numerical value to make it more accurate for diagnosis (35). When significant changes in target indicators occur in MetS patients, early monitoring and intervention can be performed, which is more valuable for diagnosing MetS combined with OA.

Frizzled 7 (*FZD7*) belongs to the G protein-coupled receptor family and is a 7-channel transmembrane cell surface receptor and a key factor in the Wnt signaling pathway (36). Frizzled proteins have a universal structure: a cysteine structural domain (CRD) that fills the extracellular space, followed by a structural domain containing seven putative transmembrane fragments (37). CRD can interact with Wnts and form a complex that reduces β-catenin phosphorylation (38). The FZD family proteins mediate the Wnt/β-catenin signaling pathway and contribute to the overall pathological progression of OA, especially in the remodeling of cartilage (39, 40). Exos derived from hADSC have been shown to contain MiR-376c-3p, which has the ability to target WNT3 or WNT9a and inhibit the Wnt/β-catenin signaling pathway, reducing synovial fibrosis and chondrocyte degradation in OA patients. In conclusion, *FZD7* can regulate cell proliferation, differentiation, migration, and tumorigenesis development by activating downstream signaling pathways through binding to Wnt ligands (41, 42).

*IRAK3*, an interleukin-1 receptor-associated kinase (IRAK) family member, is primarily expressed in immune cells such as macrophages, monocytes, dendritic cells, and epithelial cells (43). As an important regulatory protein of the TLRs/IL-1R pathway, it is involved in the inhibition of Toll-like receptor signaling. In addition, *IRAK1* and *IRAK2* can dissociate from the myosin complex, and IRAK3 inhibits it, rendering IRAK1 and *IRAK2* unable to interact with TRAF6 and thus inhibiting NF-B pathway activation, and reduces inflammation by reducing the production of pro-inflammatory factors (44). Of course, another possibility is that the MyD88-IRAK puposome interacts with *IRAK3*, which stimulates NF-B activation *via* complex formation, where both MEKK3 and TRAF6 can form complexes with *IRAK3*, which is also dependent on the NF-B pathway, to promote anti-inflammatory-related expression (45). An experiment discovered that miR-33b-3p regulated *IRAK3* and that it was effective in alleviating IL-1-induced apoptosis and inflammation. Overall, this appears to be a promising OA target (46).

*KDELR3*, a member of the KDEL family, is in charge of encoding a protein related to the endoplasmic reticulum (ER) (47). The ER influences not only the synthesis and transport of lipids and steroids but also the activity of hormones and glucose metabolism. As a result, *KDELR3* expression varies significantly between individuals, with higher levels of expression in non-atherosclerotic tissues. In addition, several studies have found that *KDELR3* is significantly aberrantly expressed in a variety of malignancies, including prostate adenocarcinoma and hepatocellular carcinoma (48, 49). As a result, we hypothesize that *KDELR3* plays a critical role in OA patients.

The HD1 structural domain of *PHC2* is close to its FCS zinc finger structural domain, which is capable of interacting with the catalytic ring structural domain of RING1B. RING1B, a core component of Polycomb Repressive Complex 1 (PRC1), can monoubiquitinate histone H2A (H2AK119ub1) and plays a catalytic role at lysine 119. It is a key regulator of the estrogen receptor alpha (ER) transcriptional process (50), through which estrogen regulates biological processes like reproductive maturation, energy homeostasis, and skeletal growth by binding to ERα (51). Most importantly, estrogen action on ERα can increase miR-140 expression levels and decrease the expression levels of matrix metalloproteinase-13 (MMP-13) in human articular chondrocytes (52). It has also been shown that *PHC2* can bind to the Vcam1 locus and act to reduce systemic immunodeficiency (53). It is reasonable to believe that *PHC2* can have an impact on the progression of OA.

*RHOB* is a Rho GTPase with a C-terminus and an N-terminus, the latter of which contains a G domain, also known as the RhoA-like domain (54). Because chromosome 2 retains *RHOB*’s genetic information and lacks the alternative *RHOB* precursor mRNA, only one *RHOB* protein sequence can be translated (55). *RHOB* can be induced by transforming growth factor beta (TGF) signaling and is immune to p53 regulation, allowing for a timely response to non-genotoxic (polysaccharide, hypoxia, inflammatory factors) and genotoxic stimuli (radiation) (56). *RHOB* deficiency inhibited pathological angiogenesis in ischemic retina patients, implying that *RHOB* alleviates symptoms by promoting the formation of lymphatic vessels following injury or in the presence of inflammation (57). Interestingly, *RHOB* also activates IL-1β (interleukin 1β), LPS (lipopolysaccharide), and TNFα (tumor necrosis factor α) in an inflammatory setting (54), contrary to previous observations. However, its mechanism of action in OA is unclear and remains to be investigated. In general, *RHOB* can regulate a variety of cellular processes, including vesicle trafficking, apoptosis, DNA repair, angiogenesis, proliferation, migration, and invasion (58).

*RNF170* is a novel E3 ubiquitin ligase that mediates the ubiquitination of the endoplasmic reticulum calcium channel sarcoglycan 1,4,5 trisphosphate receptor leading to the degradation of the sarcoglycan 1,4,5 trisphosphate receptor *via* the proteasome pathway and subsequently affecting the calcium flow and content in the endoplasmic reticulum lumen and cytoplasm. By specifically targeting TLR receptor molecules, *RNF170* mediates polyubiquitination of lysine at position 766 on the TIR domain of TLR3 (59), resulting in the degradation of TLR3 receptor molecules *via* the proteasome pathway and reducing the effect on TLR3 downstream signaling. activation of the TLR3 downstream signaling pathway, thereby inhibiting the production of inflammatory factors and type I interferons (60). A search of the GEO database revealed that *RNF170* protein expression was significantly increased in human monocytes after infection with human immunodeficiency virus (HIV) (61), and when the body’s fibroblasts underwent an immune response to cytomegalovirus infection, the expression level of *RNF170* protein in fibroblasts showed a trend of first increasing and then decreasing (62), and in general, *RNF170* is extremely important in natural immunity.

*SOX13* is a member of the SOX protein family, and its coding gene is located at 1q31.3-32.1, which is found in a wide range of cells and tissues. *SOX13* contains 604 amino acids, including three specific functional regions, namely the leucine zipper region, the glutamine-rich sequence region, and the HMG functional region (63). Its expression in three embryonic cell lineages suggests that it may be involved in various developmental processes, with expression detected in the neural tube, developing brain, kidney, liver, mesenchymal, and chondrogenic progenitor cells, and has a significant impact on sex determination, neurogenesis and endochondral bone formation (64). Innate lymphocytes, including type 1, type 2 (ILC2) and type 3 (ILC3) subpopulations, are increasingly recognized as key regulators of tissue homeostasis and inflammation through the release of cytokines (65), with NCR ILC3s producing mainly IL-17A, which is regulated by DNA binding inhibitor 2 (ID2), transcription factor 1, and SRY (sex-determining region Y)-box transcription factor 13 (Sox13) (66). Fida et al. simultaneously tested 297 cases of primary biliary sclerosis (PBC), 22 cases of autoimmune cholangitis, 29 cases of autoimmune hepatitis, and 90 patients with T1DM for *SOX13*-Ab and found a high rate of positivity (67). In addition, Sox13, as an autoimmune antigen, can also modulate T-cell specificity, and in summary, it is reasonable to suspect that *SOX13* can modulate the inflammatory response in OA.

*ZKSCAN4*, a zinc finger protein family member, localizes to chromosome 6p21-p22.1 and regulates genomic stability, stem cell generation, and telomere elongation (68, 69). It is found in a variety of tissues, including the cervix, testes, and trachea, as well as the kidneys, adrenal glands, mouth, skin, lungs, brain, spleen, uterus, liver, intestines, and muscles (70). Several authors examined European and Asian populations using genome-wide association studies (GWAS) of genes and discovered significant differences in some genes between healthy people and rheumatoid arthritis patients, especially *ZKSCAN4*, *ABCF1*, *BTN3A3*, *BRD2*, *FLOT1*, *HLA-DMA*, *HLA-G*, *HLA-F*, *HSP90AB1*, *IER3*, and *TUBB* (71). *ZKSCAN4* improves the HDM2 promoter region’s association with YY1 by modifying the HDM2 chromatin structure to promote its expression (72). HDM2 is a ubiquitin protein ligase that not only promotes the degradation but also the transcription of the tumor suppressor p53. Under hypoxia, p53 promotes cell survival by promoting the production of cellular metabolic energy in OA, inhibiting reactive oxygen species (ROS) production and ROS detoxification (73). Although there are no functional studies to validate *ZKSCAN4*, it can be speculated that it is tightly linked to OA.

Previous research has revealed that immune and inflammatory responses are present in all phases of OA. The position of inflammatory factors and cellular infiltration in the progression of OA, as well as the various manifestations of OA in different immune settings, provide a theoretical foundation for further research into the relationship between immunity and OA. Many diseases are now recognized as being influenced by the immune microenvironment, and in OA patients, both cartilage damage and repair processes involve immune cells (74). It has been established that B cells and macrophages, as the primary immune cells, are involved in cartilage damage and repair, in addition to NK cells, T cells, and DCs (75). In addition, mast cells (MC), T cells, and macrophages can be found in large numbers in the synovial tissue of OA patients. A unique pattern of immune infiltration has recently been identified, characterized by increased polarization of CD4+ T cells to activated Th1 cells and increased secretion of immunomodulatory cytokines (76). This has similarities to our findings, with OA patients having higher levels of CD8+ T cells, B-cell memory, activated dendritic cells, M0 macrophages, and eosinophils, and lower levels of Mast cells activated, T cells CD4 memory activated, M2 macrophages and Neutrophils. Above all, understanding inflammatory signaling mechanisms is critical for OA diagnosis and treatment.

## 5 Conclusion

Based on bioinformatics analysis and machine learning, we systematically identified eight related candidate genes (*FZD7*, *IRAK3*, *KDELR3*, *PHC2*, *RHOB*, *RNF170*, *SOX13*, and *ZKSCAN4*) and provided a template for the diagnosis of OA combined with MetS. We also noticed that the immune system of MetS patients with OA is out of balance, that the percentage of immune cells can be affected by the immune microenvironment, and that the screened genes could be used for clinical diagnosis and treatment.

## 6 Limitation

There are some restrictions on our research. First, despite pooling three OA datasets, the samples are still tiny, and because of the small sample size, the diagnostic value of the column line graphs is quite high. Additionally, we wanted to choose a different sample to verify the diagnostic results. The eligible ones weren’t accessible, and the Mets dataset was already small. As a result, we were unable to confirm the results. After that, the findings should be verified in a bigger study with a larger sample size. Second, even though the interactions between candidate hub genes and dysregulated immune cells warrant further study, the eight candidate hub genes are primarily concentrated in regulatory immune pathways.

## Data availability statement

The original contributions presented in the study are included in the article/**Supplementary Material**. Further inquiries can be directed to the corresponding author.

## Author contributions

All authors listed have made a substantial, direct, and intellectual contribution to the work, and approved it for publication.
