# Corrigendum: Identification of immune-associated genes in diagnosing osteoarthritis with metabolic syndrome by integrated bioinformatics analysis and machine learning

**DOI:** 10.3389/fimmu.2023.1232300

**Published:** 2023-06-27

**Authors:** Junchen Li, Genghong Wang, Xilin Xv, Zhigang Li, Yiwei Shen, Cheng Zhang, Xiaofeng Zhang

**Affiliations:** ^1^ The Graduate School, Heilongjiang University of Chinese Medicine, Harbin, China; ^2^ The Third Affiliated Hospital of Heilongjiang University of Chinese Medicine, Harbin, China; ^3^ Teaching and Research Section of Orthopedics and Traumatology, Heilongjiang University of Chinese Medicine, Harbin, China; ^4^ The Second Department of Orthopedics and Traumatology, The Second Affiliated Hospital of Heilongjiang University of Chinese Medicine, Harbin, China; ^5^ The Bone Injury Teaching Laboratory, Heilongjiang University of Chinese Medicine, Harbin, China

**Keywords:** differentially expressed genes, osteoarthritis, metabolic syndrome, machine learning, immune infiltration

In the published article, there was an error in the author list, and author Xilin Xv was erroneously excluded as corresponding author. The corrected author list appears below.

“Junchen Li 1†, Genghong Wang1†, Xilin Xv 2,3*, Zhigang Li 4, Yiwei Shen1, Cheng Zhang1 and Xiaofeng Zhang3,5*”

The authors apologize for this error and state that this does not change the scientific conclusions of the article in any way. The original article has been updated.

