# Supplementary figures and images for: Identification of immune-associated genes in diagnosing osteoarthritis with metabolic syndrome by integrated bioinformatics analysis and machine learning
Source: Front Immunol. 2023 Apr 17;14:1134412. doi: 10.3389/fimmu.2023.1134412 (PMC10150333; doi:10.3389/fimmu.2023.1134412)

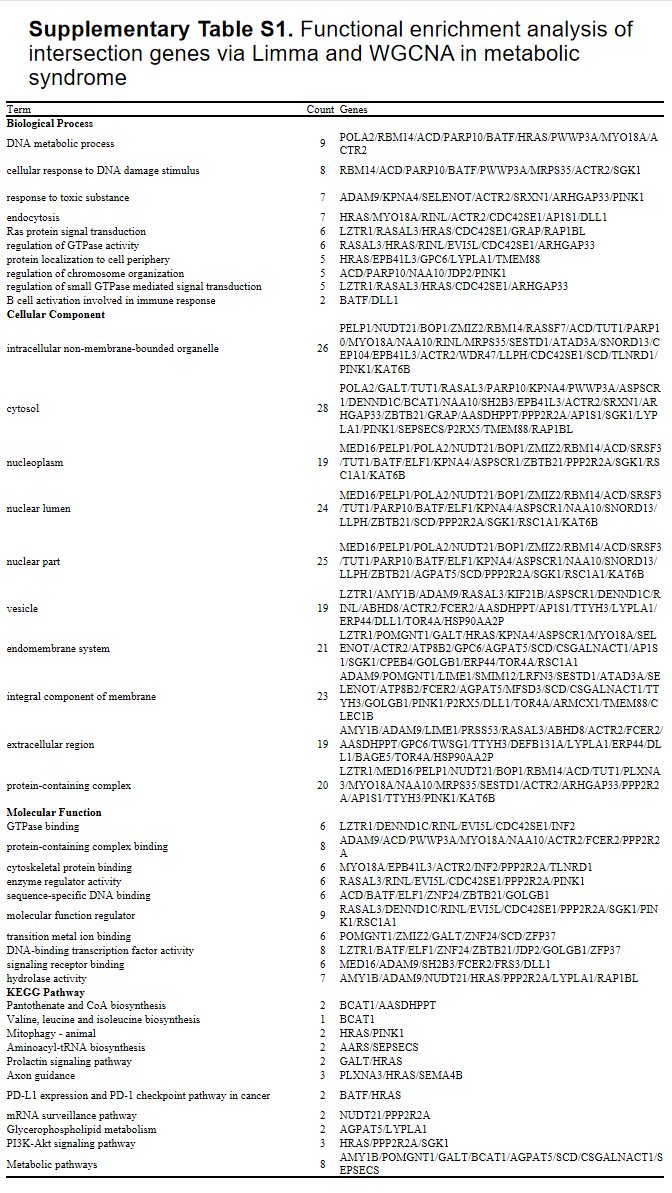

Supplement: Supplementary file 1 [file Table_1.docx]

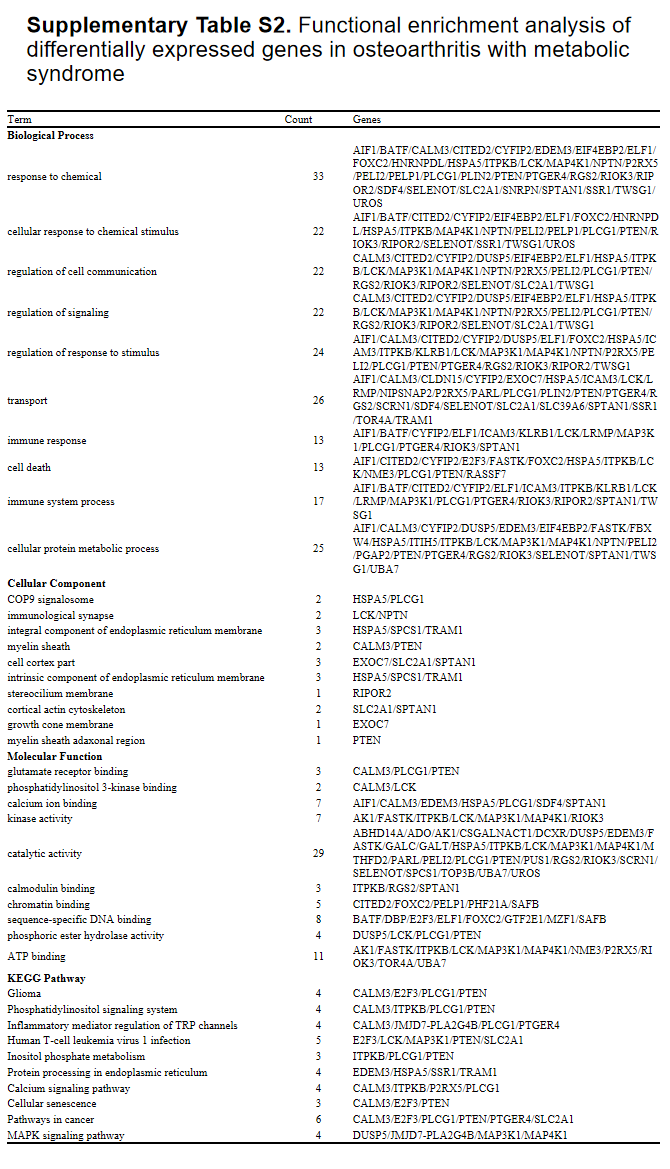

Supplement: Supplementary file 2 [file Table_2.docx]
